# Air Bubbles in the Heart: A Case of Contrast Media Injection-Induced Venous Air Embolism

**DOI:** 10.7759/cureus.8708

**Published:** 2020-06-20

**Authors:** Sohab Radwan, Scott Shepperd

**Affiliations:** 1 Internal Medicine, MedStar Washington Hospital Center, Washington DC, USA

**Keywords:** venous, air embolism, computed tomography, contrast

## Abstract

Venous air embolism (VAE) is more frequently recognized nowadays with the increased use of computed tomography (CT). It may be detected during or even after intravenous contrast media injection. A wide range of clinical manifestations exist, ranging from an incidental finding in a clinically asymptomatic patient to obstructive shock and circulatory failure. Those found incidentally are usually small and have no significant effect on circulatory physiology. Larger air emboli, however, may be potentially fatal, and therefore it is important to recognize such a phenomenon in the setting of intravenous contrast media injection.

## Introduction

Venous air embolism (VAE) during or after intravenous contrast media injection is being increasingly recognized with the more frequent use of computed tomography (CT) as a diagnostic modality in modern medicine [1]. In most cases, an air embolus within the venous system is detected incidentally [2]. Studies have shown that small volumes of air within the venous system are usually not detected clinically, but rather radiographically, as most patients are usually asymptomatic [1]. Despite having no clinical implication in such cases, this is of importance to both medical providers and radiologists, as they should be aware that such a phenomenon is common and therefore search for other sources of air is usually not required. Larger air emboli, however, when present within the venous system may have catastrophic physiological effects on circulatory hemodynamics and may potentially lead to mortality. We hereby present a case of an incidental finding of a non-fatal VAE within the heart detected on CT following intravenous contrast media injection.

## Case presentation

**Figure 1:**
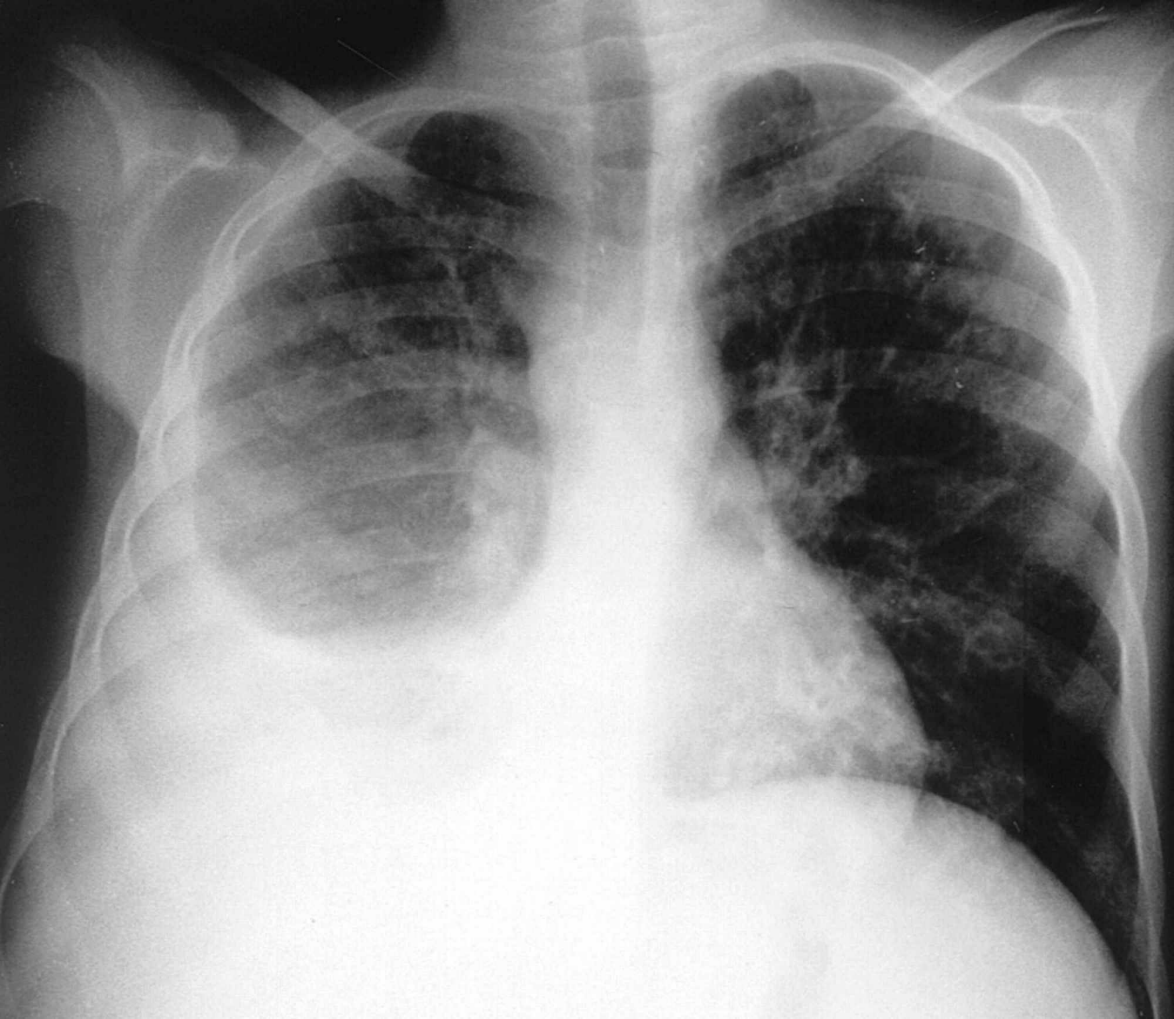
Chest radiography demonstrating right-sided pleural effusion.

A 74-year-old male with a past medical history of end-stage renal disease on intermittent hemodialysis, hypertension and type two diabetes mellitus, presented with confusion and dark-red colored sputum production of three days duration. On presentation, vital signs included blood pressure of 119/58 mmHg, heart rate of 55 beats per minute, oral temperature of 36.4 °C and respiratory rate of 27 breaths per minute with an oxygen saturation of 94% on two liters oxygen via nasal cannula. Chest X-ray revealed a right lower lung lobe opacification with blunting of the right costophrenic angle suggestive of a pleural effusion (Figure 1).

**Figure 2:**
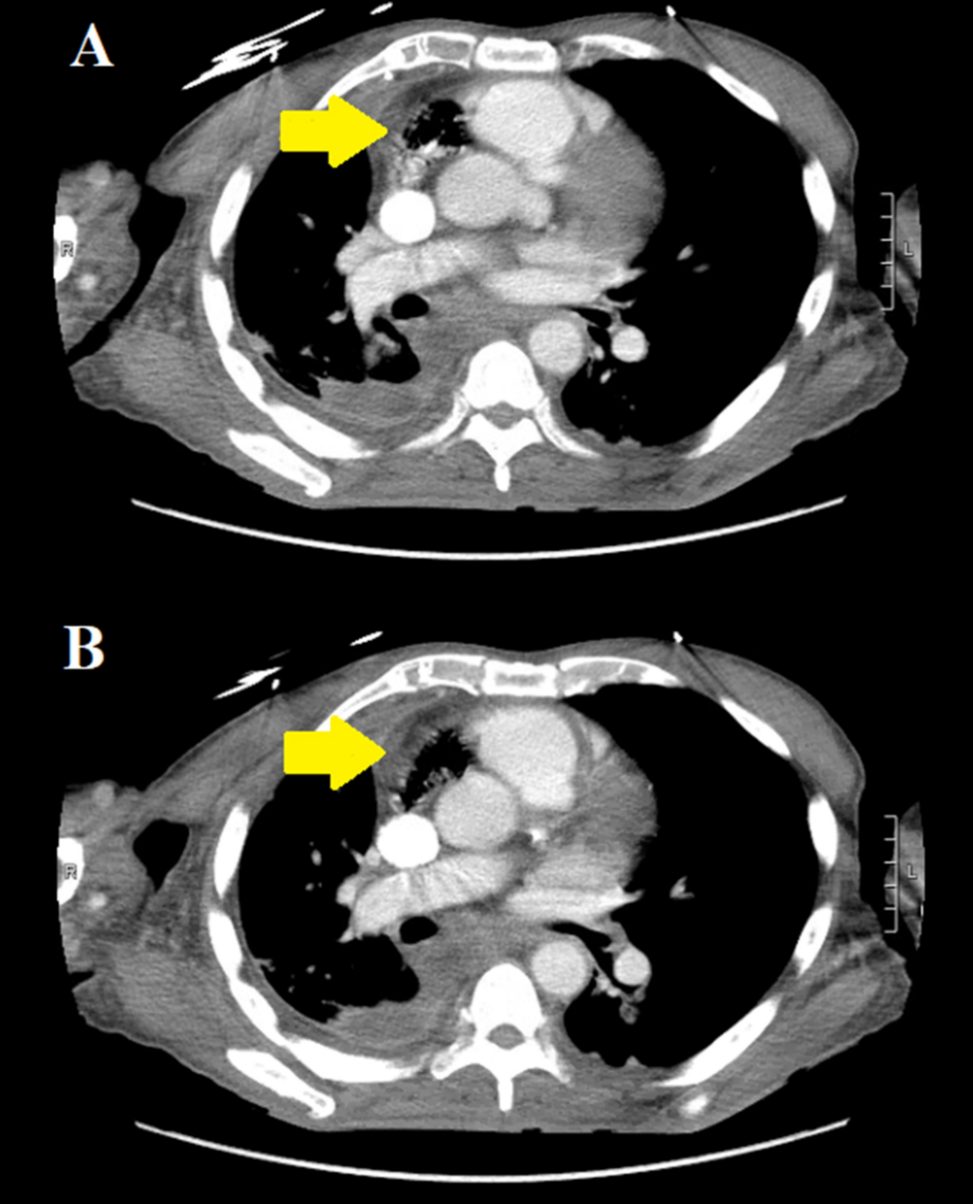
Axial chest CT with intravenous contrast demonstrating a large amount of gas in the nondependent areas of the right atrium (yellow arrows in panes A and B).

Subsequently, a chest CT scan with intravenous contrast media was obtained. This involved injection of 85 ml of iodinated contrast (Iohexol) via the right upper extremity. Chest CT demonstrated a right-sided mixed density pleural fluid collection concerning for a large empyema. Moreover, a large amount of gas was also visualized in the right atrium (Figure 2).

**Figure 3:**
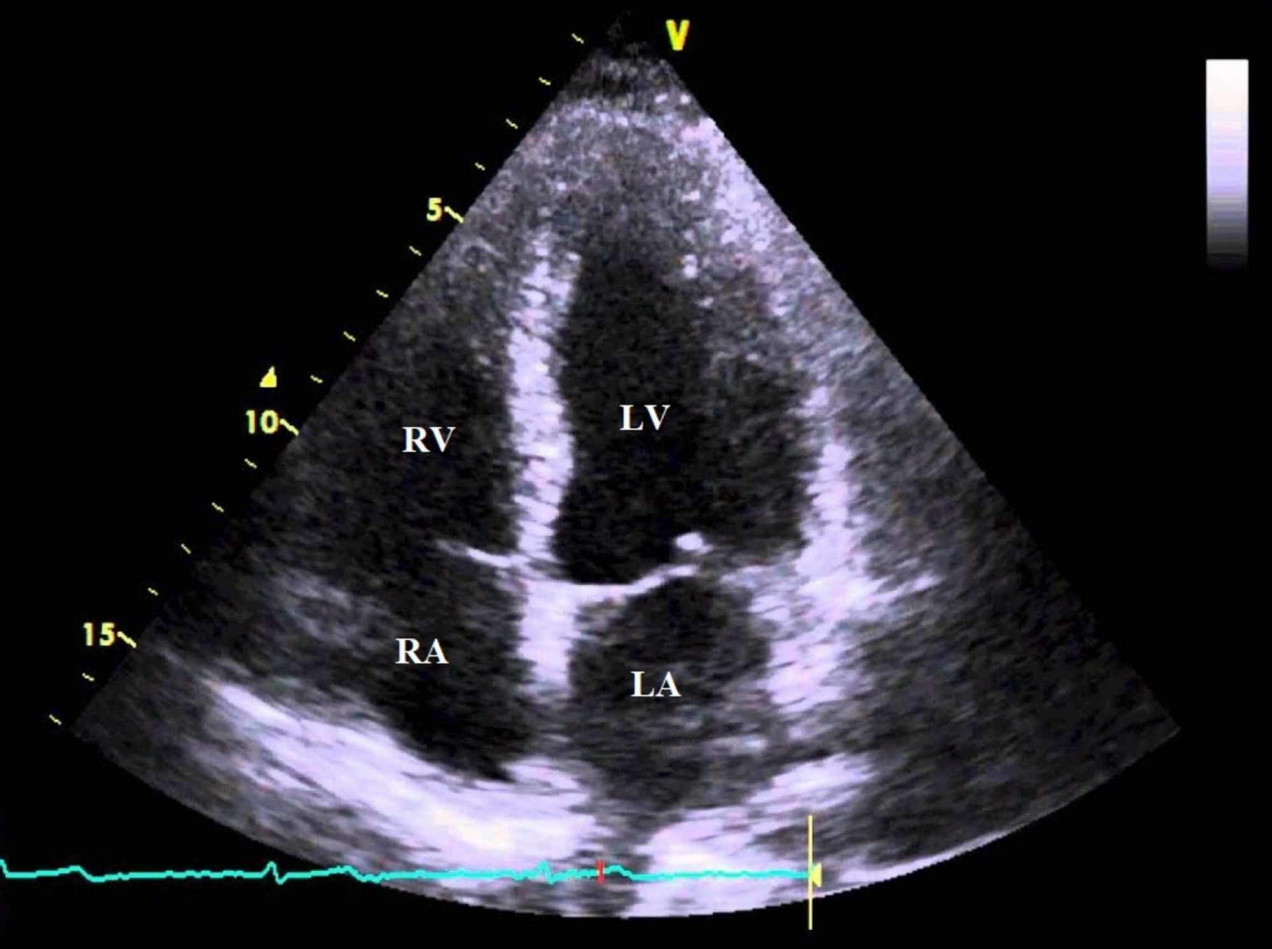
Two-dimensional transthoracic echocardiography (apical 4 chamber view) demonstrating absence of air in the right atrium. RA: right atrium; RV: right ventricle; LA: left atrium; LV: left ventricle

No fistulous tract could be identified between the heart and lung. The patient then underwent right-sided chest tube placement and was started on broad spectrum antimicrobial therapy with subsequent improvement in both mental and respiratory statuses within the next 24 to 48 hours. A two-dimensional focused transthoracic echocardiogram was obtained to investigate presence of air in the right atrium; however surprisingly, no air was visualized (Figure 3).

**Figure 4:**
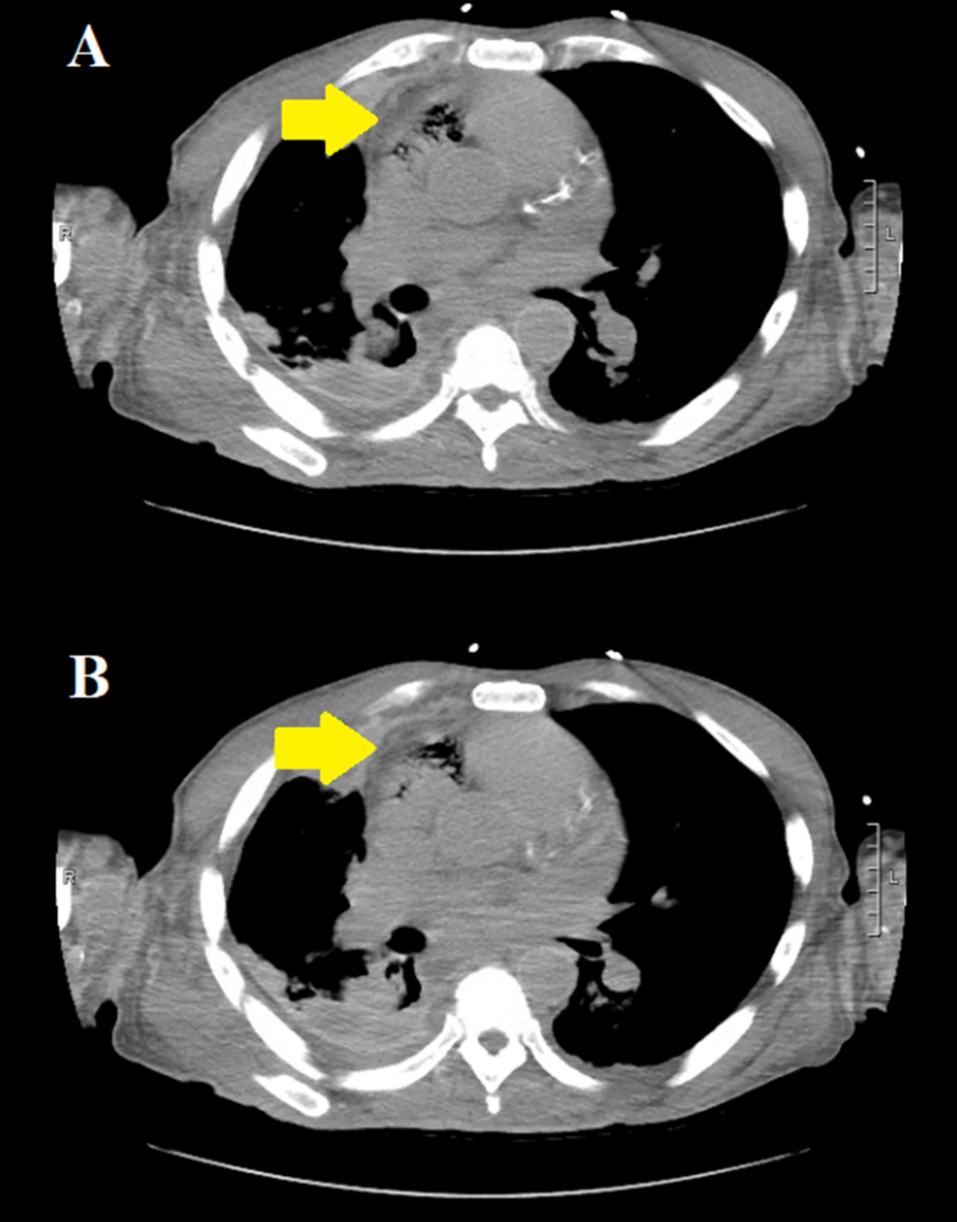
Repeat axial chest CT without intravenous contrast, 24 hours after the initial scan, demonstrating the same gas findings in the right atrium (yellow arrows in panes A and B); however of slightly less volume compared to the previous study.

A repeat chest CT scan without intravenous contrast, obtained 24 hours after the initial one, re-demonstrated the same gas findings within the anterior nondependent parts of the right atrium, but slightly less in volume in comparison to the first study (Figure 4).

**Figure 5:**
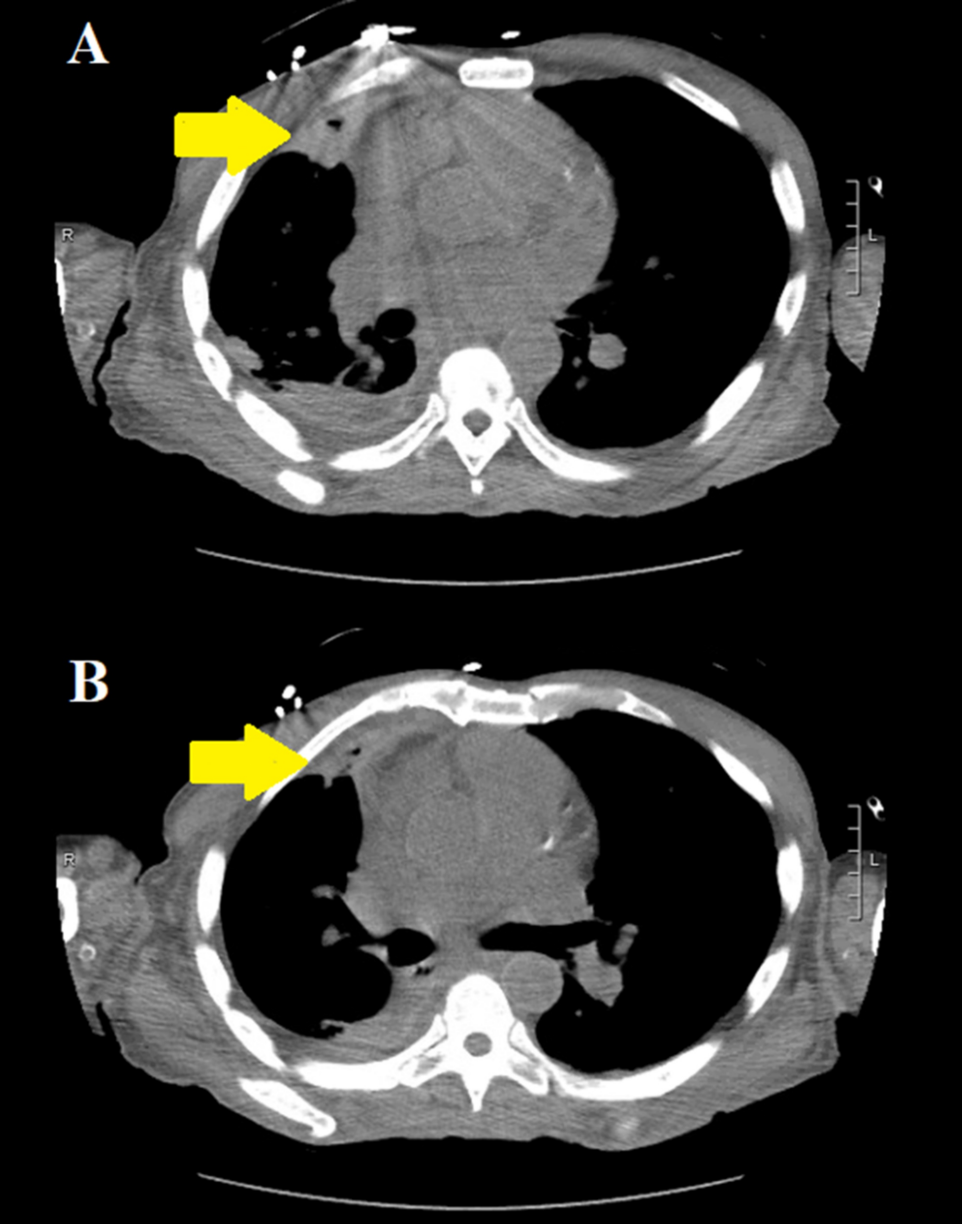
Repeat axial chest CT without intravenous contrast, four days after the second CT scan, demonstrating decreased volume and near complete resolution of gas within the right atrium (yellow arrows in panes A and B).

A third chest CT scan without intravenous contrast, obtained four days later, demonstrated continued decrease with near complete resolution of the previously noted gas within the right atrium (Figure 5).

It was favored that the visualized gas in the right atrium was secondary to forceful injection of intravenous contrast media.

## Discussion

VAE is reported to occur in 11%-23% of patients undergoing contrast enhanced CT [3]. It has been reported as well that subclinical VAE may be detected immediately following intravenous contrast media injection [4]. In terms of incidence, Groell et al. have reported an 11.7% rate of VAE following contrast enhanced CT scans versus 5.5% following non-contrasted ones [5]. Although air emboli may occur at any site within the venous system, however certain locations take preference over others. Sodhi et al. reported that the most common locations for VAE were the main pulmonary artery (60%), left brachiocephalic vein (15%), right atrial appendage (20%) and superior vena cava (5%) [2]. Predilection for such locations is mainly attributed to the buoyancy of air in a fluid medium which causes bubbles to rise to the most nondependent areas inside the vascular system [2].

Predictors of VAE have been studied and reported previously in the literature. Sodhi et al. has reported that the amount of contrast media used, intravenous cannula gauge size, flow rate and site of injection are not significantly associated with an increased incidence of VAE [2]. In terms of volume of contrast media used, a 4.2% incidence of VAE was reported in patients who received ≤50 ml of intravenous contrast media compared to 8.6% who received >50 ml, p-value 0.239 [2]. Similarly, the incidence of VAE while using an 18G cannula size was 7.8% versus 0.5% in those with a 24G cannula size, p-value 0.739 [2].

Regarding manifestations, there is a wide spectrum of clinical presentations. As mentioned earlier, VAE may present incidentally in an asymptomatic patient. Other patients may have non-specific symptoms such as dyspnea, chest pain or lightheadedness. The physiologic effects of VAE depend on the amount of air, rate of injection and the patient’s cardiopulmonary reserve [6]. It is believed that 200-300 ml of air is fatal in adult humans; however other studies have demonstrated that even 100 ml may be lethal [7,8]. Accumulation of air in the right ventricle, if significant, leads to ineffective right ventricular contraction and therefore emptying, leading ultimately to obstructive shock [6]. If the pulmonary circulation is involved, then hypoxia secondary to ventilation/perfusion mismatch ensues with consequent acute pulmonary hypertension and right ventricular failure [6]. What is more, air bubbles may enter the arterial system due to the presence of a right to left cardiopulmonary shunt such as in septal defects or a patent foramen ovale, resulting in a paradoxical air embolism.

In terms of management, no intervention is required for a small VAE that is incidentally found in otherwise clinically asymptomatic patients, as air bubbles are broken and get absorbed from the circulation [9]. Serial CT is however recommended in asymptomatic patients as a means of observation [3]. In cases of life threatening VAE, immediate management includes using the Durant’s maneuver, which involves positioning the patient in a left-lateral decubitus head-down position [10]. This maneuver helps prevent the air embolus from obstructing the right ventricular outflow tract. Additional interventions include supplemental 100% oxygen and if possible hyperbaric oxygen therapy, which has been used; however, there is insufficient evidence for its efficacy [11-13].

## Conclusions

VAE is an increasingly recognized phenomenon with the more frequent use of CT. It may be detected incidentally but should also be suspected whenever patients experience sudden onset respiratory distress following intravenous contrast media injection. For small incidentally found VAE, no active intervention is warranted if patients are hemodynamically stable, but serial CT imaging and observation is recommended.
